# Alterations of metabolites related to microbiota–gut–brain axis in plasma of colon cancer, esophageal cancer, stomach cancer, and lung cancer patients

**DOI:** 10.1515/biol-2025-1115

**Published:** 2025-05-26

**Authors:** Yuqin Zhao, Kangwei Shen, Qing Lu, Wei Huang, Xuejun Kang, Li Xie

**Affiliations:** Gynecology Department, Jiangsu Province Hospital of Chinese Medicine, Nanjing, Jiangsu, 210029, China; Key Laboratory of Child Development and Learning Science (Ministry of Education), School of Biological Science & Medical Engineering, Southeast University, Nanjing, 210096, PR China; Department of Radiotherapy, Jiangsu Province Hospital of Chinese Medicine, Nanjing, Jiangsu, 210029, China; College of Animal Science and Technology, Jinling Institute of Technology, Nanjing, 210038, PR China

**Keywords:** cancer, gut microbiota metabolites, mass spectrometry, chromatography, discrimination and diagnostic model, metabolomics

## Abstract

Co-occurring symptoms such as depression, anxiety, fatigue, and sleep disorders are frequently comorbid with cancer. The causes of these cancer-related symptom clusters are hypothesized sharing a common biological mechanism. This study explored pattern differences of some gut metabolites (glucocorticoids, short-chain fatty acids, gut microbial metabolites from tryptophan) in plasma samples from patients with four types of cancer. Metabolomics analysis was performed to indicate the differences of metabolites. Discrimination model and diagnostic model were constructed using orthogonal partial least squares discriminant analysis, and differential metabolites were screened, then receiver-operating characteristic curve analysis was performed to evaluate the performance of these models. Melatonin (MLT), indole propionic, and skatole were screened as the common differential metabolites shared by four types of cancer, indicating that the intestinal microbial metabolic pathway of tryptophan plays a key role in the occurrence and development of malignant tumors. The area under the curve values for the potential candidate biomarker predictors in univariate analysis ranged from 0.771 to 0.989, and in multivariate analysis ranged from 0.985 to 1.00. The sensitivity and specificity of the multivariable model were 94.7–100 and 96.4–100%, respectively. These biomarkers also had good performance in discriminating different pairs of cancer. The analysis of gut microbiota metabolites allows us to characterize the common metabolic characteristics of patients with various cancers. The intestinal microbial metabolic pathway of tryptophan plays a key role in the occurrence and development of malignant tumors.

## Graphical abstract

**Figure:**
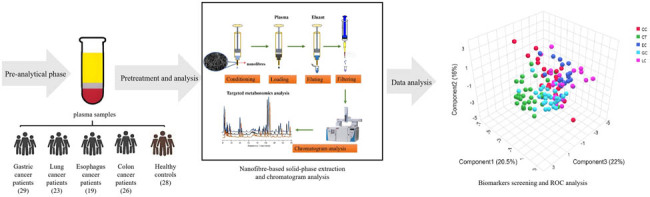

## 1 Introduction

The incidence of cancer is mainly attributed to two aspects, including genetic predisposition and exposure to environmental risk factors, but what these exact environmental factors are remains unknown [1,2]. A great deal of evidence is accumulating that psychosocial stress may be one of the factors, yet the mechanisms for this association are poorly understood [3]. Cortisol is the primary stress hormone, where gradually increasing studies have demonstrated that cortisol levels positively correlate with high mortality rate and recurrence of cancer [4,5].

In recent years, an additional environmental risk factor in cancer has drawn keen interest as the gut microbiota [6]. The intestines of mammals are home to a complex ecological system comprising thousands of bacteria, fungi, viruses, and other microorganisms [7]. A multitude of factors, including the host’s diet, hormonal levels, medication use, and environmental conditions, influence the dynamic composition of this gut microbiota, which, in turn, may contribute to the development of a variety of diseases [8]. Despite many studies on the relationship between gut microbiota and carcinogenesis, the exact mechanisms of this interaction are not very clear. Our gut microbiota is capable of producing metabolites, including a wide range of molecules with a variety of biological activities: short-chain fatty acids (SCFAs), such as acetate, propionate, and butyrate [9]; tryptophan (Trp) metabolites, such as indole (IND), indole propionic acid (IPA), melatonin (MLT), skatole (SKT), etc. [10]; amino acids, such as threonine, isoleucine, glutamine [11], and l-norvaline [12]; other substances, such as hormones, neurotransmitters, etc. [9]. Similar to human hormones, these metabolites are produced in the intestinal tract and subsequently transferred to distant sites of action through circulation. It is fluctuations of these molecules in the body that determine the beneficial or harmful role of the gut microbiota in the tumor progression, immunity, and therapy prediction by reshaping the tumor microenvironment [13]. These molecules act on target organs through blood circulation, forming the gut–organ axis, such as the microbiota–gut–liver axis or microbiota–gut–brain (MGB) axis [9].

The MGB axis refers to the bidirectional communication network linking the gut, microbiota, and brain [9]. This system enables information to be shared between the microbe and the brain, allowing the brain to communicate with the gut [14]. Cortisol receptors are expressed on various cells of the gut, indicating that cortisol can impact the MGB axis through multiple pathways, which in turn impact the composition and diversity of the gut microbiota [4,15]. Therefore, understanding the relationship between cortisol and various types of intestinal microbial metabolites is of great significance for the diagnosis, treatment, and prognosis assessment of cancer. To the best of our knowledge, few studies have examined both cortisol and representative intestinal microbial metabolites in different kinds of tumors.

This study chose to look at digestive system cancer (gastric cancer [GC], esophageal cancer [EC], and colon cancer [CC]) and lung cancer (LC) because most earlier research on the hypothalamic-pituitary-adrenal (HPA) axis and cancer focused on women with gynecological cancer, aiming to see if the HPA axis affects cancer development in a similar way across different types of cancer. Therefore, this study aimed to select several types of metabolites related to the MGB axis, including the HPA axis, for targeted metabolomics analysis. The purpose of this analysis was to investigate whether the common side effects of fatigue, insomnia, and depression observed in cancers are due to shared metabolic characteristics. Our goals were to measure the levels of two corticosteroids (cortisol and cortisone) and two groups of metabolites, SCFAs and Trp metabolites, in the blood of patients with four types of cancer (colorectal, esophageal, stomach, and LC) and healthy individuals. Metabolomics analysis was used to study how the two hormones and metabolites changed and to see if cancer types have similar biological processes. Multiple sets of potential biomarkers were identified that could be used in the diagnosis of different types of cancer.

## 2 Materials and methods

### 2.1 Study participants and sample collection

The investigation was performed with plasma samples derived from patients with GC (*n* = 29), LC (*n* = 23), EC (*n* = 19), CC (*n* = 26), and a group of healthy controls (CT) (*n* = 28). All participants in the study were acquainted with its aim and signed a written consent. Patients were recruited among patients of the Ward of Oncology Department, Jiangsu Provincial Hospital of Traditional Chinese Medicine, Nanjing, China. The sixth edition of the International Union against Cancer Classification was used to classify each cancer group, and no other coexisting cancers. All cancer patients had not received therapeutic drugs, radiation therapy, or other treatments, and blood samples were taken before treatment. The control group consisted of healthy individuals with no cancer and no chronic diseases. They were recruited among people subjected to the routine periodic medical examinations. The control group matched the cancer groups in terms of age and ethnicity (China) (Table 1). Plasma samples were obtained when the participants were waked up in the morning at about 6:00 am and immediately stored at −20℃ until being analyzed. The demographic characteristics of 97 patients and 28 age-matched health control (CT) are shown in Table 1.

**Table 1:** Demographic characteristics of the study population

Characteristics		EC	LC	Colorectal cancer	GC	Controls
No		19	23	26	28	28
Sex	Male	13	16	14	16	16
	Female	6	7	12	12	12
Age (years)	Mean	63.2	60.1	58.2	57.8	58.5


**Informed consent:** Informed consent has been obtained from all individuals included in this study.
**Ethical approval:** The research related to human use has been complied with all the relevant national regulations, institutional policies and in accordance with the tenets of the Helsinki Declaration, and has been approved by the IRB of the Affiliated Hospital of Nanjing University of Chinese Medicine by Decision no. 2023NL-KS002.

### 2.2 Cortisol and cortisone concentrations in plasma

Based on the established method for the determination of cortisol in urine [16], we briefly optimized the detection method for the analysis of target substances in plasma. Plasma cortisol was extracted using a solid phase extraction (SPE) column packed with 5 mg of polystyrene nanofiber. The simple process is as follows: the column is rinsed with 200 µL methanol and water successively to activate the extraction column. To a 100 µL of plasma, 10 µL of internal standard solution (200 ng/mL deuterated cortisol in water) was added and vortexed, the mixture was added to the small column filled with polystyrene nanofibers, and the liquid was slowly pressed out drop by drop using a barometric SPE instrument, so that the target compounds could be adsorbed on the nanofiber material. After the liquid was drained, 100 µL of methanol was added for elution in the same way. The eluted target solution was injected with 10 µL into a liquid chromatograph–mass spectrometer for the determination, and the optimized instrumental settings were presented in ref. [16] (Figure S1).

### 2.3 SCFAs concentration in plasma

Based on the SCFAs analytic method established by our research group [17], we briefly optimized the detection method for the analysis of target substances in plasma. Sample pretreatment was accomplished by a packed-fiber SPE method using 5 mg of polystyrene/polypyrrole (PS/PPy) nanofibers as the sorbent. Before SPE, 5.0 mg nanofibers were loaded into the extraction column. The nanofibers in the extraction column were activated with 200 μL of methanol and 200 μL of water, respectively. After the extraction column was activated, a plasma sample of 100 μL was added, and the fluid was pushed through the nanofibers using a barometric solid phase extractor. After passing through the column, the target substances were eluted from the column with 100 μL of eluent, which was an ethanol solution containing 0.01 mol/L hydrochloric acid. In order to remove the residue of protein precipitation contained in the eluted liquid, a gun tip column filled with 1 mg of polystyrene nanofibers was used to filter the eluted liquid again, and the filtered filtrate was collected and fed into a gas chromatography–mass spectrometer for the determination. Other instruments and experimental conditions remained unchanged (Figure S2).

### 2.4 Trp metabolites concentration in plasma

Based on the analytic method for Trp metabolites established by our research group [18], we briefly optimized the detection method for the analysis of target substances in plasma. About 100 μL of plasma sample was added with concentrated HCl (hydrochloric acid: plasma = 1:1, v/v). The acidified samples were centrifuged at a rotational speed of 12,000 rpm for 3.0 min and then the supernatant was separated. Before SPE, columns packed with 5.0 mg of PS/PPy nanofibers were activated with 100 μL of methanol and 100 μL of water, respectively. After the extraction column was activated, 100 μL of the treated sample supernatant was added, and then the sample liquid was pressurized through the nanofibers using a barometric solid phase extractor. After passing through the column, the target substance adsorbed was eluted from the column with 100 μL of eluent containing methanol and 0.2 mol/L sulfuric acid (95:5). Finally, the eluent was injected into a liquid chromatograph for the determination (Figure S3).

### 2.5 Statistical analysis

Urinary creatinine concentrations were measured to correct urinary metabolite concentrations and control the influence of urinary volume fluctuations. Data were analyzed by Statistical Product and Service Solutions (IBM SPSS 27.0.1), and Mann–Whitney test was used to conduct descriptive statistics on creatinine adjusted urinary target compound levels of subjects. Mean ± SD was calculated to determine the overall gut microbiota metabolite profiles. The *p*-values <0.05 were used to assess statistical significance. Natural ln transformation of all target compounds was performed using GraphPad Prism 10.1.2 for mapping purposes. The metabolomics dataset was processed using MetaboAnalyst 6.0. All missing values were replaced by the average of each variable within the group, and a natural logarithmic transformation of the variable was performed. Principal component analysis (PCA) and orthogonal partial least squares discriminant analysis (OPLS-DA) were used to visualize metabolic changes between HC and cancer groups. MetaboAnalyst 6.0 was used for biomarker analysis of differential metabolites, including classic univariate receiver-operator characteristic (ROC) analysis and multivariate ROC analysis. In biomarkers analysis, the sample first passed normalization, data transformation, and data scaling to make the normalized data normally distributed, and biomarker analysis was then performed. Finally, pathway analysis was performed and visualized using the MetaboAnalyst 6.0 software package.

## 3 Results

### 3.1 Characterizing the biochemical composition of plasma in four type of cancer patients

**Figure 1:**
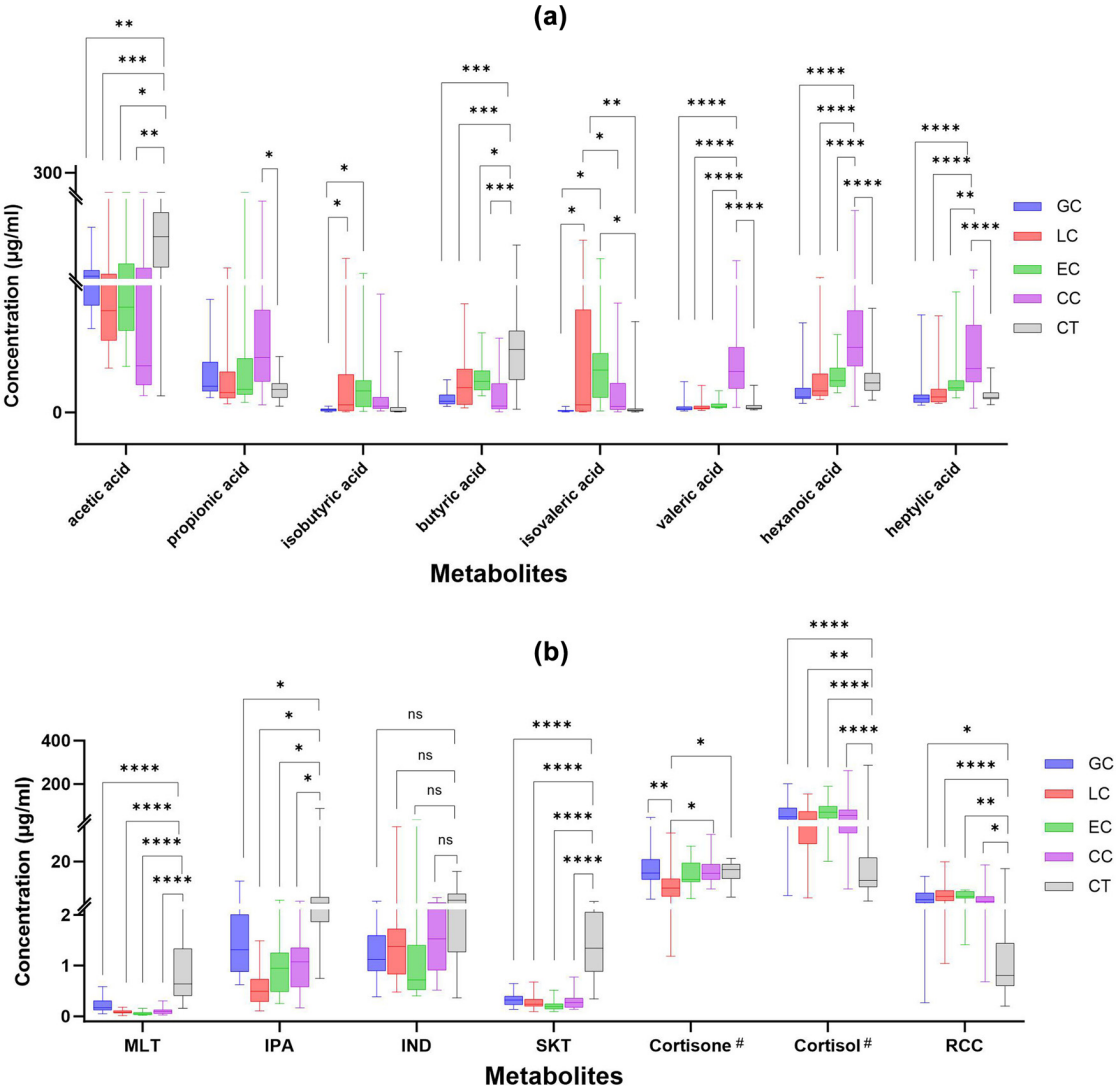
Univariate multivariate analysis of metabolite concentrations of patient groups and health control group. (a) metabolites of short-chain fatty acid; (b) metabolites of Indoleamine and corticosteroid; Boxplots illustrate the upper quartile, median (central transverse line), and lower quartile, with whiskers indicating the maximum and minimum values. # Concentration unit of cortisol and cortisone in plasma is ng/mL. *p < 0.05, **p < 0.01, ***p < 0.001, ****p < 0.0001, ns *p* > 0.05. GC, gastric cancer; LC, lung cancer; EC, esophageal cancer; CC, colon cancer; CT, healthy controls. IND, indole, IPA, indole propionic acid, MLT, melatonin, SKT, skatole.

A total of 14 targeted metabolites in each sample of cancers and health controls were determined, including acetic acid, propionic acid, isobutyric acid, butyric acid, isovaleric acid, valeric acid, hexanoic acid, heptylic acid, MLT, IPA, IND, and SKT, cortisone, and cortisol. Since the ratio of cortisol to cortisone (RCC) characterizes the enzyme 11β-HSD1 that catalyzes the transformation of these two corticosteroids *in vivo*, RCC was also included as an indicator in the data calculation. All metabolite concentrations are as shown in Figure 1.

As shown in Figure 1a, the concentrations of acetic acid and butyric acid across the cancers were significantly lower than those of the control group (*p* < 0.05); on the contrary, propionic acid, isobutyric acid, and isovaleric acid were mostly higher than those of the control group; however, the increases were not significant in most disease groups. The concentrations of valeric acid, caproic acid, and heptanoic acid were significantly higher in colorectal cancer than that of other types of cancers and the control group (*p <* 0.01).

As shown in Figure 1b, the concentrations of MLT, IPA, and SKT were significantly lower in all types of cancers than those of the control group (*p <* 0.05), while the concentrations of IND were not significantly different. The concentrations of cortisol and RCC across cancers were significantly different from those of the control group, with the former significantly higher (*p <* 0.01) and the latter significantly lower (*p <* 0.05), and the changes of cortisone level in all groups were not very obvious.

### 3.2 Correlations between metabolites in plasma

**Figure 2:**
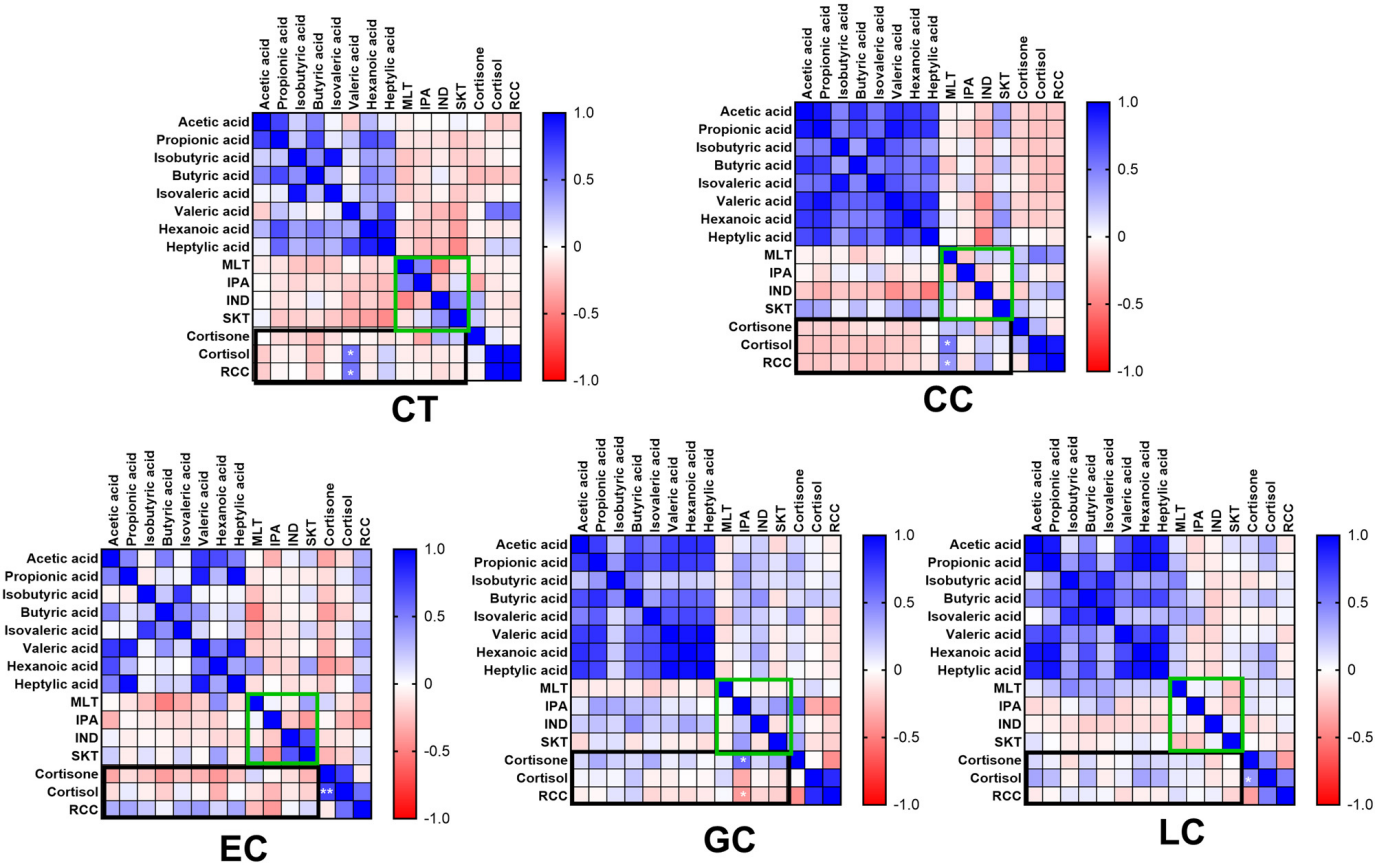
Correlation map calculated using Pearson’s correlation coefficient of metabolites in blood plasma. Pearson’s correlation coefficient is reported with color gradient, and blue color indicates a positive association, while red indicates a negative association. Only the significant correlations (*p*  <  0.05) of two corticosteroids are illustrated with asterisks (*p  <  0.05, **p  <  0.001). GC, gastric cancer; LC, lung cancer; EC, esophageal cancer; CC, colon cancer; CT, healthy controls. IND, indole, IPA, indole propionic acid, MET, melatonin, SKT, skatole.

The correlations of the 14 compounds within five groups are presented in Figure 2. Pearson’s correlation was used to test for the correlation of the 14 metabolites measured in plasma. Compared with the control group, the correlations between metabolites were changed, except for the EC group, the other three groups showed a significantly enhanced positive correlation between SCFAs. The relationship between SCFAs and Trp metabolites were changed from negative correlation in normal group to slightly positive correlation for some metabolites in cancer groups. And the correlations of cortisol, cortisone, and their ratio (RCC) with SCFAs and Trp metabolites were also changed, and the changes were different in each type of cancer. Except for individual material pairs, the general trend toward enhanced positive correlation was observed.

### 3.3 Multivariate statistical analysis for control and cancer subjects

**Figure 3:**
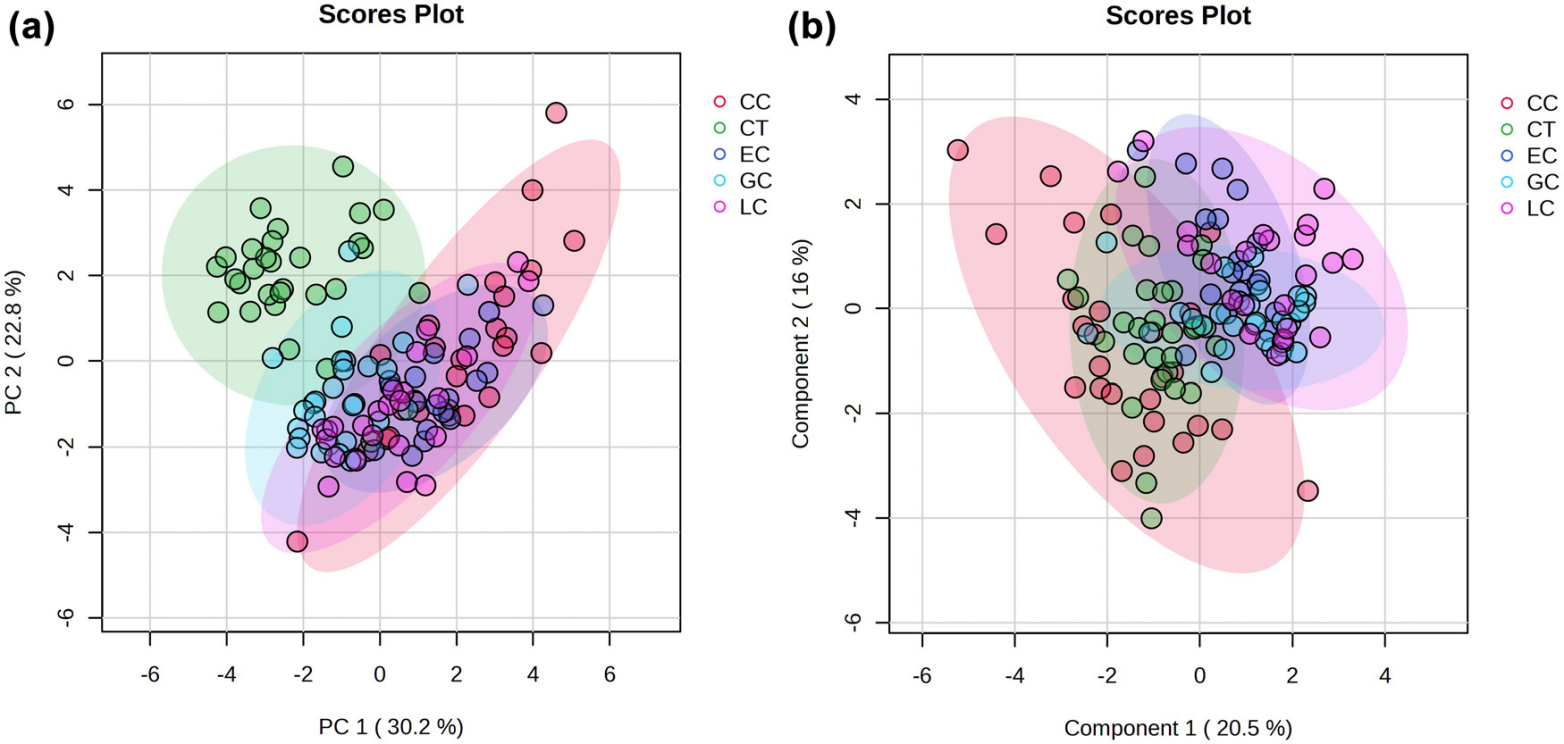
(a) PCA score plots and (b) PLS-DA score plots based on the plasma samples from five patient groups with different cancers in comparison with the healthy control group (GC, gastric cancer; LC, lung cancer; EC, esophageal cancer; CC, colon cancer; CT, healthy controls).

The absolute quantitative data of 14 accurately measured data were grouped into four cancers and a control group and brought into MetaboAnalyst 6.0 for statistical analysis and mapping. PCA was carried out to generate an overview of the variations between groups. Figure 3a shows the PC1 vs PC2 score plot for all the samples. The PCA distribution did not exhibit any significant trend or difference across the groups. In order to determine the metabolites that contributed to the differences between five groups, partial least squares discriminant analysis (PLS-DA) was employed to examine the data; however, the score plots among the five groups indicated that the groups could not be distinctly differentiated (Figure 3b).

**Figure 4:**
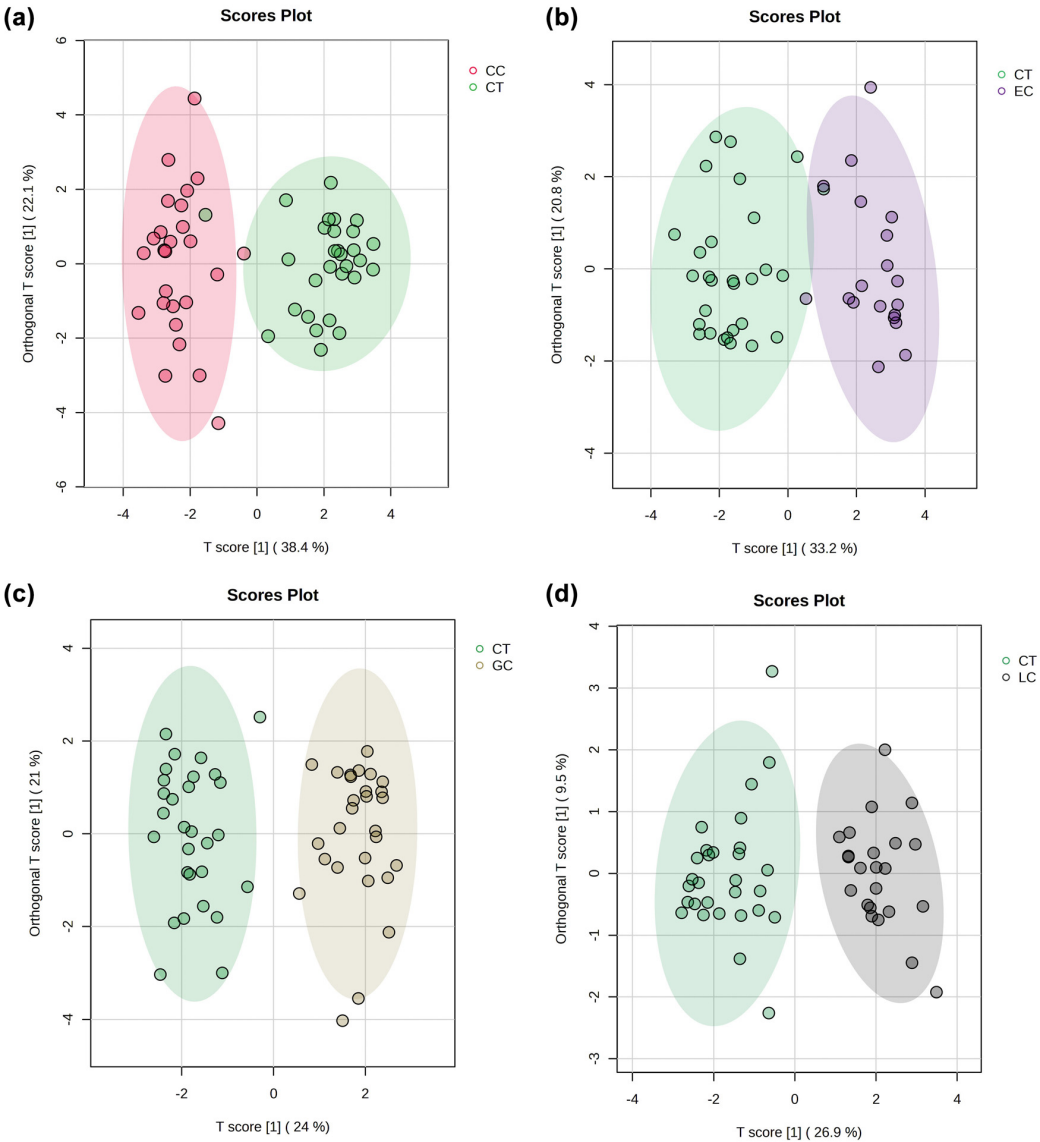
OPLS-DA score plots based on the plasma samples from each patient group in comparison with healthy control group: (a) colon cancer, (b) EC, (c) GC, and (d) LC.

However, if OPLS-DA was carried out to generate an overview of the variations between control and each cancer group. The data revealed significant differences between the normal and disease groups (Figure 4a–d). The subsequent parameters of cross validation and permutation tests met the requirements (Q2, 0.752–0.827; R2Y, 0.845–0.868), indicating that the model was reliable in predicting performance. The results showed that there were significant metabolic differences between cancers and the healthy control group.

### 3.4 Differential metabolites and biomarker analysis

**Figure 5:**
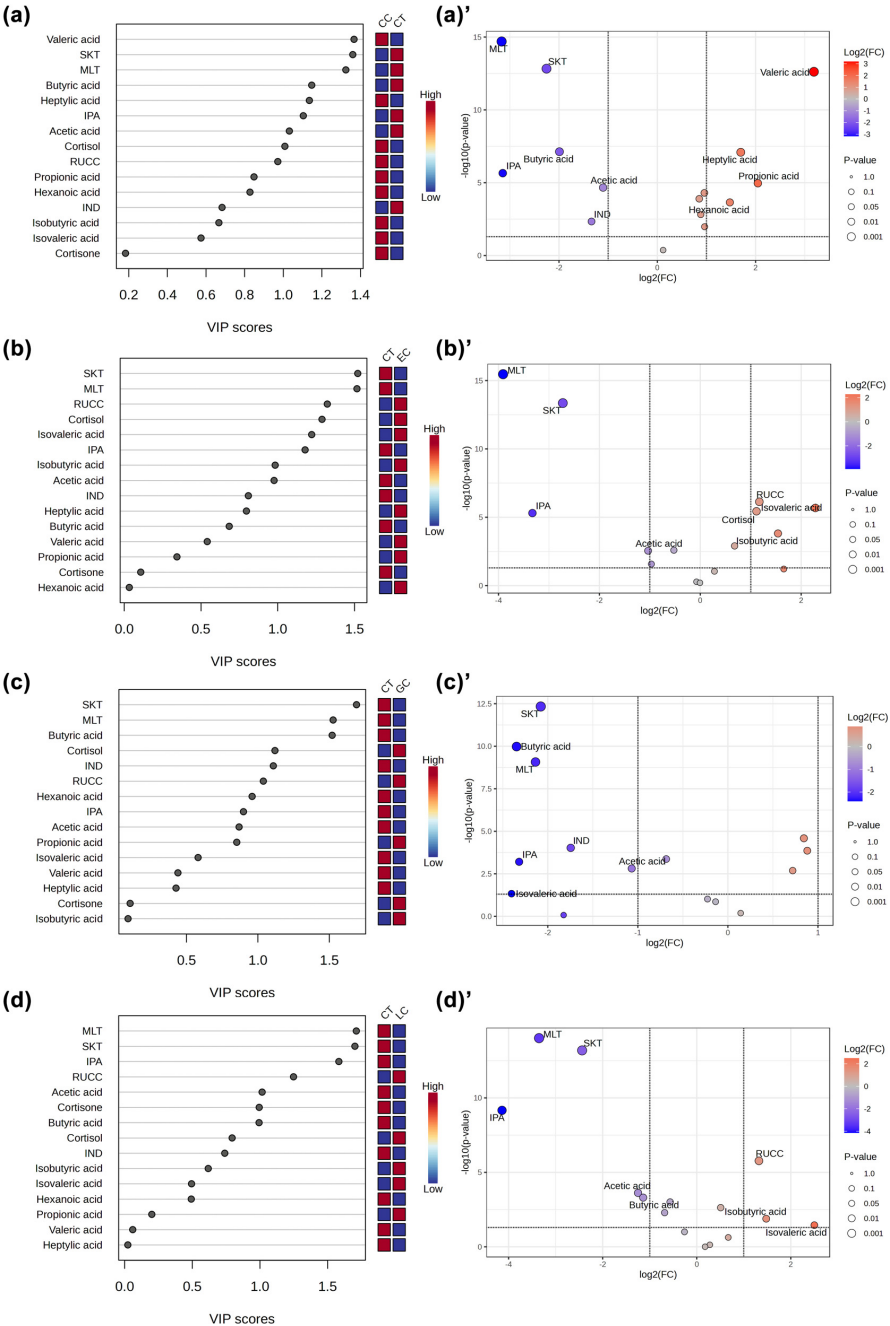
Important features identified by the OPLS-DA and volcanic map of metabolites: (a, a’) CC, (b, b’) EC, (c, c’) GC, and (d d’) LC (IND, indole, IPA, indole propionic acid, MET, melatonin, SKT, skatole).

The variable importance in projection (VIP) value was calculated according to the established OPLS-DA model. Among the 14 endogenous metabolites detected, metabolites with *p* values <0.05 serve as potential biomarkers. The observed differences were attributable to significant differences between the concentrations of the metabolites represented in the VIP scores (Figure 5a–d). The |log2FC| ≥ 1 and VIP ≥ 1 were used as the selection criteria for deferential metabolites. Figure 5a’–d’ down-regulated (blue dot) and up-regulated metabolites (red dot). The differential metabolites between cancers and control group are presented in Table 2. It was found that MLT, SKT, and IPA were down-regulated in all cancer groups (except IND replaces IPA in GC group), acetic acid and butyric acid down-regulated, while heptylic acid and valeric acid up-regulated in CC groups, isovaleric acid up-regulated in EC, and RCC and cortisol up-regulated only in EC group.

**Table 2:** Differential metabolites found in the metabolomic analysis and AUC values of the metabolites by ROC curve analysis of the normal group and the disease groups

Group	Compound	FC	log2(FC)	VIP	Type	Univariate (AUC)	Multivariate (AUC)	Sensitivity (%)	Specificity (%)
CC	IPA	0.1131	−3.1443	1.104	Down	0.893	0.999	96.2	100
	MLT	0.1113	−3.1674	1.324	Down	0.989			
	SKT	0.2096	−2.2541	1.360	Down	0.967			
	Acetic acid	0.4654	−1.1033	1.032	Down	0.793			
	Butyric acid	0.2515	−1.9914	1.148	Down	0.861			
	Heptylic acid	3.2437	1.6976	1.135	Up	0.875			
	Valeric acid	9.0989	3.1857	1.367	Up	0.952			
EC	IPA	0.0996	−3.3282	1.178	Down	0.929	0.996	94.7	96.6
	MLT	0.0665	−3.9097	1.516	Down	0.996			
	SKT	0.1512	−2.7252	1.521	Down	0.985			
	Isovaleric acid	2.9094	1.5407	1.221	Up	0.853			
	RCC	2.2523	1.1714	1.323	Up	0.895			
	Cortisol	2.1647	1.1142	1.288	Up	0.895			
GC	IND	0.2982	−1.7455	1.108	Down	0.771	0.985	89.7	96.4
	MLT	0.2274	−2.1366	1.527	Down	0.909			
	SKT	0.2368	−2.0784	1.690	Down	0.950			
	Butyric acid	0.1966	−2.3468	1.520	Down	0.935			
LC	IPA	0.0567	−4.14	1.584	Down	0.985	1.00	100	100
	MLT	0.0979	−3.353	1.712	Down	0.988			
	SKT	0.1846	−2.4378	1.702	Down	0.981			
	Acetic acid	0.4209	−1.2486	1.016	Down	0.825			

CC: colon cancer; EC: esophageal cancer; GC: stomach cancer; LC: lung cancer; IPA: indole propionic acid; MET: melatonin; SKT: skatole; RCC: ratio of cortisol to cortisone.

ROC curve analysis was performed to further characterize the predictive value of differential metabolites in differentiating tumor patients from healthy controls. The predictive utility of the ROC curve was measured by the area under the curve (AUC). An AUC of 0.5 or <0.5 for the metabolite indicates that the difference between the groups were not significant, and AUC close to 1 is considered a perfect discriminant test. As shown in Table 2, the AUC values for the predictors in univariate analysis ranged from 0.771 to 0.989, and in multivariate analysis ranged from 0.985 to 1.00. The sensitivity and specificity of the multivariable model were 94.7–100 and 96.4–100%, respectively.

In addition to differentiating between patients with each type of cancer and the controls, pairings between the four cancer groups were also identified by multivariate ROC analysis. The accuracies of all discriminant analyses using the concentrations of differential metabolites as explanatory variables were better than 55% (Table 3).

**Table 3:** Multivariate ROC analysis between different pairs of cancer

Group	Compound	Another cancer	Multivariate (AUC)	Sensitivity (%)	Specificity (%)
CC	IPA, MLT, SKT, acetic acid, butyric acid, heptylic acid, valeric acid	EC	0.947	88.5	100
		GC	0.952	88.5	96.4
		LC	0.962	88.5	95.7
EC	IPA, MLT, SKT, isovaleric RCC, cortisol	CC	0.721	78.9	69.2
		GC	0.917	89.5	96.4
		LC	0.667	78.9	56.5
GC	IND, MLT, SKT, butyric acid	CC	0.774	82.1	69.2
		EC	0.912	85.7	89.5
		LC	0.944	85.7	90.0
LC	IPA, MLT, SKT, acetic acid	CC	0.639	69.2	78.3
		EC	0.652	57.9	78.3
		GC	0.930	82.6	82.1

CC: colon cancer; EC: esophageal cancer; GC: stomach cancer; LC: lung cancer; IPA: indole propionic acid; MET: melatonin; SKT: skatole; RCC: ratio of cortisol to cortisone.

### 3.5 Altered metabolic pathways

**Figure 6:**
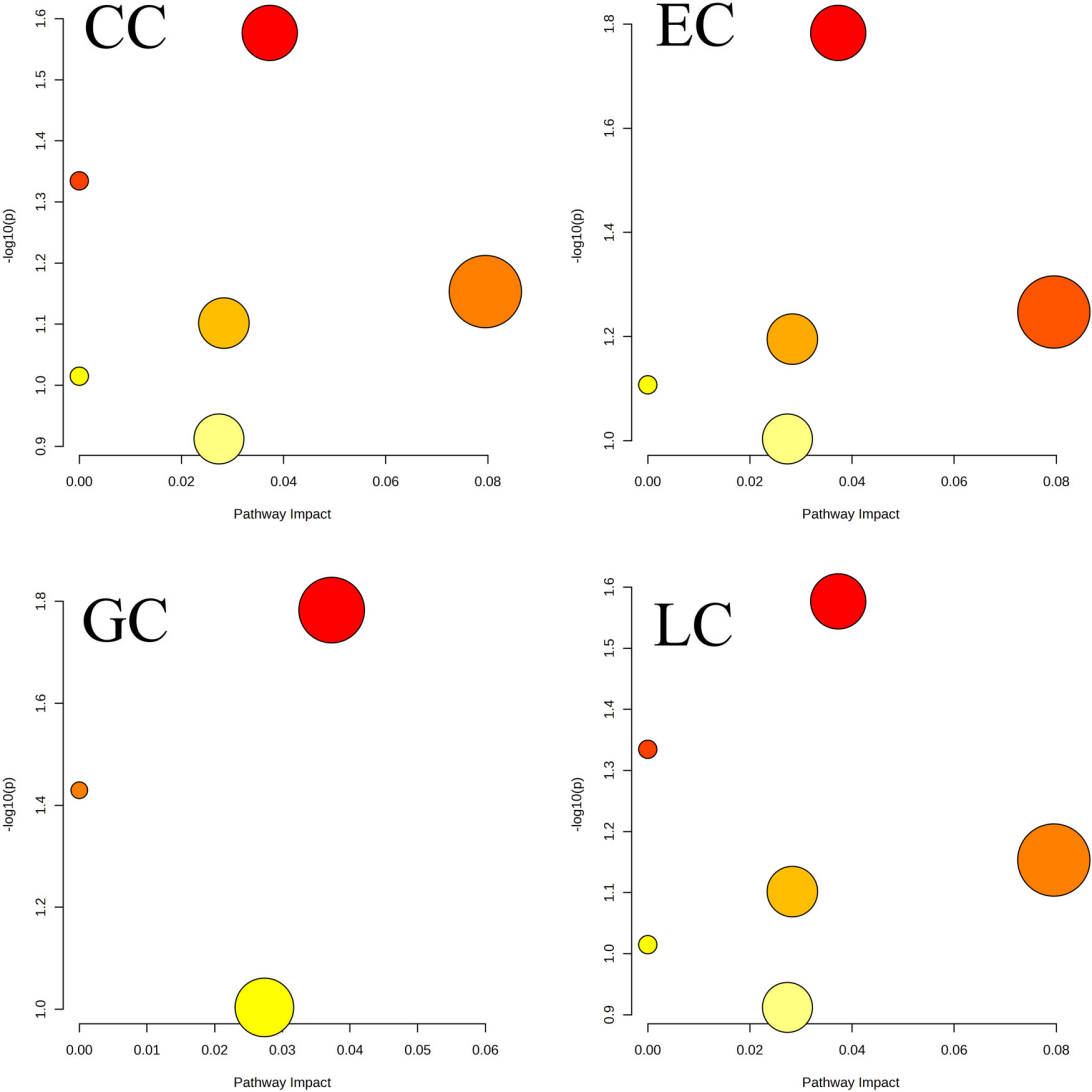
Metabolome view of pathway changes observed by differential metabolites in plasma of four types of cancer patients. GC, gastric cancer; LC, lung cancer; EC, esophageal cancer; CC, colon cancer.

When we analyzed pathways with differential metabolites (shown in Table 2) for cancers vs control group, several metabolic pathways showed cancer-related changes. As shown in Figure 6, changes in steroid hormone biosynthesis and Trp metabolism were significant and common across cancers, and changes in pyruvate metabolism, glycolysis, or gluconeogenesis were also significant except GC.

## 4 Discussion

### 4.1 Determination methods and application

Cancer-related fatigue [19], sleep difficulties [20], depressive symptoms [21], and cognitive impairment [22] are common side effects of cancer. The often co-occurring and interdependent nature of these symptoms suggests that a common underlying mechanism may exist [23]. The discovery of shared mechanisms of action pathways may help develop new strategies to manage multiple symptoms.

In this study, three classes of metabolites related to MGB axis were detected in plasma samples from four tumor groups and healthy control group, so as to investigate the changing characteristics of the three classes of indicators in different tumor groups and explore whether there may be some common biological mechanisms shared in different tumor groups, and evaluate its possibility as multiple clinical markers and prognostic evaluation indicators. To achieve the goal, we used the electrospinning technology to prepare nanofiber solid-phase extraction materials that can interact with target substances through hydrogen bonds, electrostatics, hydrophobicity, coordination, host–guest, etc. Combined with the nanofiber-based solid-phase extraction established by our research group, analytical methods for three classes of metabolites related to MGB axis in plasma samples was established based on slightly modified analytical methods that have been reported [16–18]. These methods were applied to the detection of metabolic profiles of flora-related metabolites in plasma samples from four groups of patients with cancer. Based on metabolomics analysis, it could be possible to explore the changing trends and patterns of intestinal flora metabolic-related target substances in the human body after being affected by cancer, and to explore the possible mechanisms of flora metabolic disorder in cancer patients.

### 4.2 Cortisol and cortisone alternations

Multiple evidence suggests that the immunosuppressive effect of cortisol may reduce cancer immune surveillance, promote immune escape, and induce carcinogenic mutations [24]. Cortisol can induce obesity and insulin resistance, and is associated with an increased risk of a range of malignant tumors [25]. In addition, chronic stress from exposure to environmental factors leads to an increased risk of cancer [26].

A recent review of HPA axis dysfunction in cancer reports that most studies report increased baseline cortisol or hyperactive cortisol response, while a few report decreased baseline cortisol or cortisol response in cancers. However, the authors also point out some limitations of the listed studies, such as the heterogeneity of assay methods and sampling protocols, and they conclude that a standardized approach is needed to study the mechanisms of HPA disorders and their health outcomes in order to develop appropriate tools to diagnose and manage HPA dysfunction in cancers [27].

In this study, the results of endogenous cortisol and cortisone in four groups of cancer patients showed that the plasma cortisol levels were significantly increased compared with the control group, and the cortisol/cortisone ratios were increased, indicating that the activity of 11β-HSD1 may be enhanced and the activity of 11β-HSD2 may be weakened. This suggests that there may be common mechanisms of change for various types of cancers in glucocorticoid metabolism, and it is necessary to evaluate patients’ cortisol levels before and after cancer diagnosis and treatment, respectively.

### 4.3 Trp metabolite alternations

MLT is an active metabolite of Trp produced by host’s own cells in the serotonergic pathway in a circadian rhythm under the control of various enzymes [28]. MLT exerts considerable functional versatility with antioxidant, oncostatic, anti-aging, anti-stress, and immunomodulatory properties [29]. Decreased melatonin levels increase the risk of certain cancers such as breast cancer, endometrial cancer, colorectal cancer, non-Hodgkin lymphoma, and prostate cancer [30]. There is current interest in exogenous MLT as a potential anti-proliferative agent in some cancers [30].

This study found that compared with the control group, the concentrations of MLT across the cancers were significantly lower than those in the control group (*p* < 0.05). Many previous studies have typically focused on a single type of cancer, for example, several studies have found that MLT concentrations in cancer patients are lower than in healthy controls, including prostate cancer [31], breast cancer [32], cervical cancer [32], LC [33,34], and colorectal cancer [35]. In this work, the MLT level in all types of cancers was detected simultaneously, the results showed that they were similar to those in individual cancer studies. Therefore, significantly reduced plasma MLT levels may be common in cancers and could potentially serve as a biomarker for early detection. Further research is warranted to explore the underlying mechanisms contributing to these diminished levels and their implications for cancer progression and treatment outcomes.

In addition to MLT, other Trp metabolites also have an impact on cancer development, such as IND, IPA, and SKT are active metabolites of Trp produced by gut microbes [28]. Sári et al.’s study on breast cancer found that 3-indoleacetic acid has anti-tumor properties, and IPA reduces the proportion of cancer stem cells and the proliferation, movement, and metastasis of cancer cells [36]. Sári also points out that IPA supplementation can reduce the invasion of the surrounding tissue by the primary tumor, the number of metastases, cell movement, and blood exudation. IND derivatives support the survival of breast cancer patients, and their levels are down-regulated as the disease progresses. Our test results showed that IPA and SKT were significantly lower in the plasma of four cancer groups than in the control group, which is similar to the results reported in the literature, suggesting that supplementation with certain Trp metabolites may be beneficial for the treatment of cancers.

### 4.4 SCFAs alternation

SCFAs are a class of metabolites produced by gut microbes, which can reduce inflammation, maintain intestinal barrier, mediate colonization resistance of intestinal pathogens, and enhance host health. Studies have shown that SCFAs can prevent and improve various malignant tumors, such as adenocarcinoma [37] and colorectal cancer [38].

Acetic acid inhibits the proliferation of human cancer cell lines by reducing glycolysis [39]. The potential benefits of the combination of acetate and propionate in tumor growth inhibition have raised concerns [40]. Butyrate is the most studied SCFA that inhibits the progression and proliferation of cancer cells by regulating metabolic, endocrine, and immune functions [41]. Butyrate and propionate can enhance the effectiveness of chemotherapy drugs by increasing tumor sensitivity or enhancing anti-tumor immune response [42].

Acetate, butyrate, and propionate have been widely studied due to their high abundance, while SCFAs, isobutyrate, valerate, and isovalerate, in less abundance, have received less attention. Recent studies have shown that isobutyric acid can inhibit the growth of colorectal cancer cells and synergistically improve the efficacy of anti-PD-1 immunotherapy [43].

Zhu et al. collected stool samples from breast cancer patients to detect SCFAs, and found that compared with the control group, the contents of acetic acid and butyric acid were lower, while the contents of isobutyric acid and valeric acid were higher (*p* < 0.05) [12].

This study found that the concentrations of acetic acid and butyric acid in the plasma samples of the four tumors were significantly lower than those of the control group (*p <* 0.05), and propionic acid, isobutyric acid, and isovaleric acid were mostly higher than those of the control group. However, the results were not significant in some cancer groups. These results were consistent with Zhu’s study, although the types of tumors studied were different. For valeric acid, hexanoic acid, and heptylic acid, these molecules with more carbon atoms, the changes of these SCFAs in colorectal cancer samples were distinctive, and the concentrations were significantly higher than that of other cancer groups and control group (*p <* 0.01).

### 4.5 Relationships between the metabolites

There was also a correlation between the three types of metabolites investigated in this study. For example, under stress conditions, glucocorticoids reduce the synthesis of MLT in the pineal gland, and the production of MLT at night can only be activated under low stress conditions [44,45].

MLT is also thought to counteract the immunosuppressive effect of glucocorticoids, on the one hand up-regulating the expression of MLT receptors on the surface of lymphoid organs cells, and on the other hand decreasing the sensitivity of immunoactive cells to glucocorticoids [46].

Xu et al. found that MLT is associated with its metabolites. In addition, MLT and its metabolites are also associated with cortisol and several steroid hormones upstream and downstream of cortisol metabolism [47].

Dalile et al. administered a colonic SCFAs mixture (acetate, propionic acid, butyrate) to healthy participants, and the results showed a significant reduction in cortisol levels and stress responses. This study supports the mechanistic role of SCFAs in the human MGB axis, particularly in relation to stress reactivity dominated by the HPA axis where cortisol is the marker [48].

In this study, it was observed that the relationships between the metabolites were changed by comparing the healthy group with the patient groups, and most of Pearson’s correlation coefficients were increased in the same group of metabolites, such as in SCFAs (except EC group) and glucocorticoids groups, while in the group of Trp metabolites, Pearson’s correlation coefficients were mostly reduced. The correlations between metabolites of different classes were mostly changed slightly from negative to positive between SCFAs and metabolites of Trp. The changes in Pearson’s correlation coefficients between glucocorticoids and the other two groups of metabolites were different in different disease groups, but the changes were not significant.

### 4.6 Potential biomarkers

In this study, 14 target compounds were analyzed targeting the MGB axis, and four groups of potential candidate biomarkers were found on the basis of screening differential metabolites. These biomarkers had good performance in discriminating between disease and healthy groups, and most of them showed acceptable effects in discriminating different pairs of cancer.

### 4.7 Metabolic pathway alternations

Tumor cells increase their metabolic rate to maintain rapid growth through “metabolic reprogramming,” which is most often caused by increased glycolysis. Pyruvate is the end product of the anaerobic glycolytic energy production pathway, and the anaerobic decomposition of pyruvate produces lactic acid or ethanol as a byproduct. Glycolytic enzymes and products have been shown to be closely associated with tumor progression, including proliferation, invasion, metastasis, autophagy, and immune escape. Diagnostic imaging such as positron emission tomography and magnetic resonance spectrum, and the detection of glycolytic enzymes have been developed to provide evidence for accurate diagnosis and disease management in cancer patients [49]. This study also provides information on abnormal glycolysis and pyruvate metabolism in cancer patients through the detection of several metabolites in plasma.

The association between stress and cortisol reactivity is well known. Exposure to stress causes HPA axis to activate and release glucocorticoids from the adrenal cortex. As the end product of the HPA axis, cortisol is widely used as a marker of neuroendocrine stress responses [50]. A recent literature suggests that glucocorticoids produced by immune and tumor cells within tumors have been shown to support tumor immune escape [24]. The HPA axis is an important regulator of the immune system, and its potential inhibitory effect on immune function may affect the occurrence and progression of cancer. Previous studies on the relationship between glucocorticoids and cancer have mostly focused on female tumor patients. Adding cortisol to cultured breast cancer cells *in vitro* has been shown to enhance or inhibit the growth of breast cancer cells. A small number of studies have reported elevated cortisol in breast cancer patients, and the amount raised is positively correlated with the severity of the cancer [51]. We found that plasma glucocorticoid production and metabolism were active in patients with EC, GC, LC, and colorectal cancer, suggesting that this may be a potential mechanism for the co-occurrence and development of malignant tumors.

In addition, we found down-regulated plasma Trp metabolites and an imbalance in Trp metabolism that is common in patients with four types of cancer. It has been reported that dysregulated Trp metabolism promotes tumor growth and immune escape by creating immunosuppressive tumor microenvironment [52].

### 4.8 Shortcomings and advantages

This study had some limitations, such as a small sample size and lack of clinical personal information, such as IBM and cancer stage, which made more detailed clinically relevant analyses difficult to carry out. In addition, our samples were all from Han Chinese derived from a single-center, which may have influenced the results and limited external validity. Confounding factors such as diet and drug use were not strictly controlled. Further studies are needed to expand the study to include different ethnic, sample sizes to provide more accurate and meaningful results.

The advantage of this study was that the quantitative determination method was relatively sensitive and accurate, and the sample pretreatment with nanofiber materials was conducive to eliminate the possible sample matrix effect in the usual detection, making the results more reliable.

## 5 Conclusion

Many previous studies have typically focused on a single type of cancer. This approach screened valuable gut microbiota markers for different types of cancer, contributing to a deeper understanding of the biology, pathogenesis, and treatment strategies of specific cancers. However, this approach also has certain limitations, as it may overlook commonalities and differences between different cancer types.

In this study, metabolomic analysis targeting SCFAs, four Trp metabolites, and two glucocorticoids was performed using Packed-nanofiber SPE coupled with chromatography–mass method. MLT, IPA, and SKT were screened as the common differential metabolites shared by four types of cancer indicating that the intestinal microbial metabolic pathway of Trp plays a key role in the occurrence and development of malignant tumors. In addition, some SCFAs can also be used as potential biomarkers for diagnostic models. All four disease groups showed significant increases in cortisol and RCC levels. These results suggest that paying close attention to the changes in these three types of metabolites will play a potential role in the diagnosis and treatment of cancer. Four groups of potential candidate biomarkers were found on the basis of screening differential metabolites for discriminating disease and healthy groups, and these biomarkers also play a role in models that classify different cancers.

## Supplementary Material

Supplementary Figure

## References

[1] Blackadar CB. Historical review of the causes of cancer. World J Clin Oncol. 2016;7:54–86. 10.5306/wjco.v7.i1.54 PMC4734938 26862491

[2] Urbaniak C, Gloor GB, Brackstone M, Scott L, Tangney M, Reid G. The microbiota of breast tissue and its association with breast cancer. Appl Environ Microbiol. 2016;82:5039–48. 10.1128/AEM.01235-16 PMC4968547 27342554

[3] Giudice A, Aliberti SM, Barbieri A, Pentangelo P, Bisogno I, D’Arena G, et al. Potential mechanisms by which glucocorticoids induce breast carcinogenesis through Nrf2 inhibition. Front Biosci-Landmark. 2022;27:223. 10.31083/j.fbl2707223 35866405

[4] errera RA, Deshpande K, Martirosian V, Saatian B, Julian A, Eisenbarth R, et al. Cortisol promotes breast-to-brain metastasis through the blood–cerebrospinal fluid barrier. Cancer Rep. 2022;5:e1351. 10.1002/cnr2.1351 PMC9124512 33635590

[5] Hsiao FH, Jow GM, Kuo WH, Huang CS, Lai YM, Liu YF, et al. The partner’s insecure attachment, depression and psychological well-being as predictors of diurnal cortisol patterns for breast cancer survivors and their spouses. Stress Amst Neth. 2014;17(2):169–75. 10.3109/10253890.2014.880833 24393005

[6] Ruo SW, Alkayyali T, Win M, Tara A, Joseph C, Kannan A, et al. Role of gut microbiota dysbiosis in breast cancer and novel approaches in prevention, diagnosis, and treatment. Cureus. 2021;13:e17472. 10.7759/cureus.17472 PMC8405251 34513524

[7] Álvarez J, Fernández Real JM, Guarner F, Gueimonde M, Rodríguez JM, Saenz De Pipaon M, et al. Gut microbes and health. Gastroenterol Hepatol (Engl Ed). 2021;44:519–35. 10.1016/j.gastrohep.2021.01.009 33652061

[8] Wargo JA. Modulating gut microbes. Science. 2020;369:1302–3. 10.1126/science.abc3965 32913089

[9] Álvarez-Mercado AI, Del Valle Cano A, Fernández MF, Fontana L. Gut microbiota and breast cancer: the dual role of microbes. Cancers. 2023;15:443. 10.3390/cancers15020443 PMC9856390 36672391

[10] Tsvetikova SA, Koshel EI. Microbiota and cancer: host cellular mechanisms activated by gut microbial metabolites. Int J Med Microbiol. 2020;310:151425. 10.1016/j.ijmm.2020.151425 32423739

[11] Plaza-Diaz J, Álvarez-Mercado AI. The interplay between microbiota and chemotherapy-derived metabolites in breast cancer. Metabolites. 2023;13:703. 10.3390/metabo13060703 PMC10301694 37367861

[12] Zhu Q, Zai H, Zhang K, Zhang X, Luo N, Li X, et al. L-Norvaline affects the proliferation of breast cancer cells based on the microbiome and metabolome analysis. J Appl Microbiol. 2022;133:1014–26. 10.1111/jam.15620 35543360

[13] Zhou Y, Han W, Feng Y, Wang Y, Sun T, Xu J. Microbial metabolites affect tumor progression, immunity, and therapy prediction by reshaping the tumor microenvironment. Int J Oncol. 2024;65:73. 10.3892/ijo.2024.5661 PMC11173369 38847233

[14] Alpert O, Begun L, Issac T, Solhkhah R. The brain–gut axis in gastrointestinal cancers. J Gastrointest Oncol. 2021;12(Suppl 2):S301–10. 10.21037/jgo-2019-gi-04 PMC8343079 34422394

[15] Priego-Parra BA, Remes-Troche JM. Bidirectional relationship between gastrointestinal cancer and depression: the key is in the microbiota–gut–brain axis. World J Gastroenterol. 2024;30(48):5104–10. 10.3748/wjg.v30.i48.5104 PMC11612697 39735265

[16] Li C, Zhang Z, Liu X, Shen K, Gu P, Kang X. Simultaneous quantification of cortisol and cortisone in urines from infants with packed-fiber solid-phase extraction coupled to HPLC–MS/MS. J Chromatogr B. 2017;1061–1062:163–8. 10.1016/j.jchromb.2017.07.012 28735224

[17] Zhao R, Chu L, Wang Y, Song Y, Liu P, Li C, et al. Application of packed-fiber solid-phase extraction coupled with GC–MS for the determination of short-chain fatty acids in children’s urine. Clin Chim Acta. 2017;468:120–5. 10.1016/j.cca.2017.02.016 28237548

[18] Wei L, Kang X. Packed-nanofiber solid-phase extraction coupled with high-performance liquid chromatography fluorescence for determining gut microbiota–host cometabolites and indoleamines in human urine. Separations. 2024;11:153.

[19] Goto T, Saligan LN. Mechanistic insights into behavioral clusters associated with cancer-related systemic inflammatory response. Curr Opin Support Palliat Care. 2024;18(3):161–7. 10.1097/SPC.0000000000000706 38814249

[20] Lowery-Allison AE, Passik SD, Cribbet MR, Reinsel RA, O’Sullivan B, Norton L, et al. Sleep problems in breast cancer survivors 1–10 years posttreatment. Palliat Support Care. 2018;16:325–34. 10.1017/S1478951517000311 28508735

[21] Saeki Y, Sumi Y, Ozaki Y, Hosonaga M, Kenmotsu Y, Onoe T, et al. Proposal for managing cancer-related insomnia: a systematic literature review of associated factors and a narrative review of treatment. Cancer Med. 2024;13(22):e70365. 10.1002/cam4.70365 PMC11586868 39584650

[22] Aspelund SG, Halldorsdottir T, Agustsson G, Sigurdardottir Tobin HR, Wu LM, Amidi A, et al. Biological and psychological predictors of cognitive function in breast cancer patients before surgery. Support Care Cancer Off J Multinatl Assoc. Support Care Cancer. 2024;32(1):88. 10.1007/s00520-023-08282-5 38185720

[23] Amidi A, Wu LM. Circadian disruption and cancer- and treatment-related symptoms. Front Oncol. 2022;12:1009064. 10.3389/fonc.2022.1009064 PMC9650229 36387255

[24] Schwarzlmueller P, Triebig A, Assié G, Jouinot A, Theurich S, Maier T, et al. Steroid hormones as modulators of anti-tumoural immunity. Nat Rev Endocrinol. 2025;01102:2. 10.1038/s41574-025-01102-2 40128599

[25] Nead KT, Sharp SJ, Thompson DJ, Painter JN, Savage DB, Semple RK, et al. Evidence of a causal association between insulinemia and endometrial cancer: a Mendelian randomization analysis. J Natl Cancer Inst. 2015;107(9):djv178. 10.1093/jnci/djv178 PMC4572886 26134033

[26] Bratborska AW, Piotrowski I. The impact of yoga practice on cortisol levels in breast cancer patients – a comprehensive review. Oncol Clin Pract. 2024;20(6):420–7.

[27] Kanter NG, Cohen‐Woods S, Balfour D, Burt MG, Waterman AL, Koczwara B. Hypothalamic–pituitary–adrenal axis dysfunction in people with cancer: a systematic review. Cancer Med. 2024;13(22);e70366. https://onlinelibrary.wiley.com/doi/10.1002/cam4.70366. 10.1002/cam4.70366 PMC11579619 39569439

[28] Konopelski P, Mogilnicka I. Biological effects of indole-3-propionic acid, a gut microbiota-derived metabolite, and its precursor tryptophan in mammals’ health and disease. Int J Mol Sci. 2022;23:1222. 10.3390/ijms23031222 PMC8835432 35163143

[29] Simonneaux V, Ribelayga C. Generation of the melatonin endocrine message in mammals: a review of the complex regulation of melatonin synthesis by norepinephrine, peptides, and other pineal transmitters. Pharmacol Rev. 2003;55(37):325–95. 10.1124/pr.55.2.2 12773631

[30] Jung-Hynes B, Huang W, Reiter RJ, Ahmad N. Melatonin resynchronizes dysregulated circadian rhythm circuitry in human prostate cancer cells: melatonin and the circadian clock. J Pineal Res. 2010;49:60–8. 10.1111/j.1600-079X.2010.00767.x PMC3158680 20524973

[31] Lozano-Lorca M, Olmedo-Requena R, Rodríguez-Barranco M, Redondo-Sánchez D, Jiménez-Pacheco A, Vázquez-Alonso F, et al. Salivary melatonin rhythm and prostate cancer: CAPLIFE study. J Urol. 2022;207:565–72. 10.1097/JU.0000000000002294 34694161

[32] Bartsch C, Bartsch H, Jain AK, Laumas KR, Wetterberg L. Urinary melatonin levels in human breast cancer patients. J Neural Transm. 1981;52:281–94. 10.1007/BF01256753 6801199

[33] Chang WP, Lin CC. Relationships of salivary cortisol and melatonin rhythms to sleep quality, emotion, and fatigue levels in patients with newly diagnosed lung cancer. Eur J Oncol Nurs. 2017;29:79–84. 10.1016/j.ejon.2017.05.008 28720269

[34] Hu S, Shen G, Yin S, Xu W, Hu B. Melatonin and tryptophan circadian profiles in patients with advanced non-small cell lung cancer. Adv Ther. 2009;26:886–92. 10.1007/s12325-009-0068-8 19802530

[35] Khoory R, Stemme D. Plasma melatonin levels in patients suffering from colorectal carcinoma. J Pineal Res. 1988;5:251–8. 10.1111/j.1600-079x.1988.tb00651.x 3404397

[36] Sári Z, Mikó E, Kovács T, Jankó L, Csonka T, Lente G, et al. Indolepropionic acid, a metabolite of the microbiome, has cytostatic properties in breast cancer by activating AHR and PXR receptors and inducing oxidative stress. Cancers. 2020;12:2411. 10.3390/cancers12092411 PMC7565149 32854297

[37] Sivaprakasam S, Prasad PD, Singh N. Benefits of short-chain fatty acids and their receptors in inflammation and carcinogenesis. Pharmacol Ther. 2016;164:144–51. 10.1016/j.pharmthera.2016.04.007 PMC4942363 27113407

[38] Zhang Z, Cao H, Song N, Zhang L, Tai J. Long-term hexavalent chromium exposure facilitates colorectal cancer in mice associated with changes in gut microbiota composition. Food Chem Toxicol. 2020;138:111237. 10.1016/j.fct.2020.111237 32145354

[39] Sahuri-Arisoylu M, Mould RR, Shinjyo N, Bligh SWA, Nunn AVW, Guy GW, et al. Acetate induces growth arrest in colon cancer cells through modulation of mitochondrial function. Front Nutr. 2021;8:588466. 10.3389/fnut.2021.588466 PMC8081909 33937302

[40] Tang Y, Chen Y, Jiang H, Nie D. The role of short-chain fatty acids in orchestrating two types of programmed cell death in colon cancer. Autophagy. 2011;7:235–7. 10.4161/auto.7.2.14277 21160278

[41] Portincasa P, Bonfrate L, Vacca M, De Angelis M, Farella I, Lanza E, et al. Gut microbiota and short chain fatty acids: implications in glucose homeostasis. Int J Mol Sci. 2022;23:1105. 10.3390/ijms23031105 PMC8835596 35163038

[42] Al-Qadami GH, Secombe KR, Subramaniam CB, Wardill HR, Bowen JM. Gut microbiota-derived short-chain fatty acids: impact on cancer treatment response and toxicities. Microorganisms. 2022;10:2048. 10.3390/microorganisms10102048 PMC9612155 36296324

[43] Murayama M, Hosonuma M, Kuramasu A, Kobayashi S, Sasaki A, Baba Y, et al. Isobutyric acid enhances the anti-tumour effect of anti-PD-1 antibody. Sci Rep. 2024;14:11325. 10.1038/s41598-024-59677-1 PMC11101641 38760458

[44] Da Silveira Cruz-Machado S, Tamura EK, Carvalho-Sousa CE, Rocha VA, Pinato L, Fernandes PAC, et al. Daily corticosterone rhythm modulates pineal function through NFκB-related gene transcriptional program. Sci Rep. 2017;7:2091. 10.1038/s41598-017-02286-y PMC5437068 28522814

[45] Fernandes PA, Tamura EK, D’Argenio-Garcia L, Muxel SM, Da Silveira Cruz-Machado S, Marçola M, et al. Dual effect of catecholamines and corticosterone crosstalk on pineal gland melatonin synthesis. Neuroendocrinology. 2017;104:126–34. 10.1159/000445189 26954684

[46] Singh AK, Haldar C. Melatonin modulates glucocorticoid receptor mediated inhibition of antioxidant response and apoptosis in peripheral blood mononuclear cells. Mol Cell Endocrinol. 2016;436:59–67. 10.1016/j.mce.2016.07.024 27452798

[47] Xu W, Cui Y, Guo D, Wang W, Xu H, Qiao S, et al. UPLC-MS/MS simultaneous quantification of urinary circadian rhythm hormones and related metabolites: application to air traffic controllers. J Chromatogr B. 2023;1222:123664. 10.1016/j.jchromb.2023.123664 37040674

[48] Dalile B, Vervliet B, Bergonzelli G, Verbeke K, Van Oudenhove L. Colon-delivered short-chain fatty acids attenuate the cortisol response to psychosocial stress in healthy men: a randomized, placebo-controlled trial. Neuropsychopharmacology. 2020;45:2257–66. 10.1038/s41386-020-0732-x PMC7784980 32521538

[49] Qiao Q, Hu S, Wang X. The regulatory roles and clinical significance of glycolysis in tumor. Cancer Commun Lond Engl. 2024;44(7):761–86. 10.1002/cac2.12549 PMC11260772 38851859

[50] Ahabrach H, El Mlili N, Mafla-España MA, Cauli O. Hair cortisol concentration associates with insomnia and stress symptoms in breast cancer survivors. Int J Psychophysiol. 2023;191:49–56. 10.1016/j.ijpsycho.2023.07.006 37532197

[51] Luecken LJ, Compas BE. Stress, coping, and immune function in breast cancer. Ann Behav Med. 2002;24(4):336–44. 10.1207/S15324796ABM2404_10 12434945

[52] Zhang HL, Zhang AH, Miao JH, Sun H, Yan GL, Wu FF, et al. Targeting regulation of tryptophan metabolism for colorectal cancer therapy: a systematic review. RSC Adv. 2019;9(6):3072–80. 10.1039/c8ra08520j PMC9060217 35518968

