# Supplementary material for: Alterations of metabolites related to microbiota–gut–brain axis in plasma of colon cancer, esophageal cancer, stomach cancer, and lung cancer patients
Source: Open Life Sci. 2025 May 26;20(1):20251115. doi: 10.1515/biol-2025-1115 (PMC12120410; doi:10.1515/biol-2025-1115)
Supplement: Supplementary Figure [file biol-2025-1115-sm.pdf]

Supplementary material

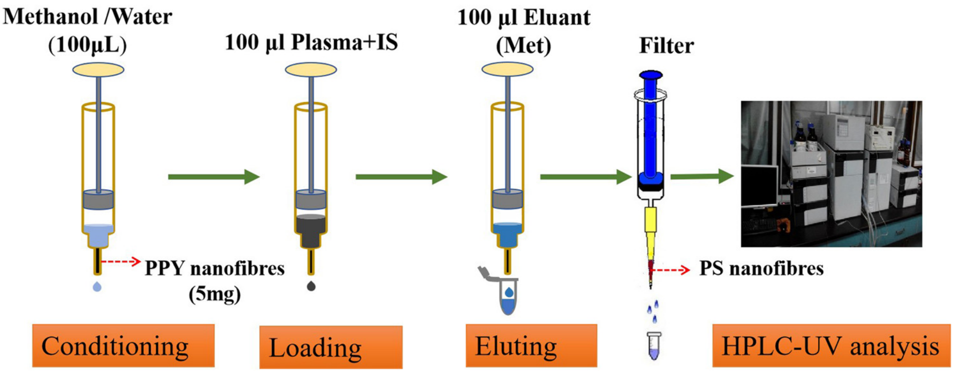

Figure S1: Schematic flow chart of PFSPE procedure for the determination of cortisol and cortisone in plasma.

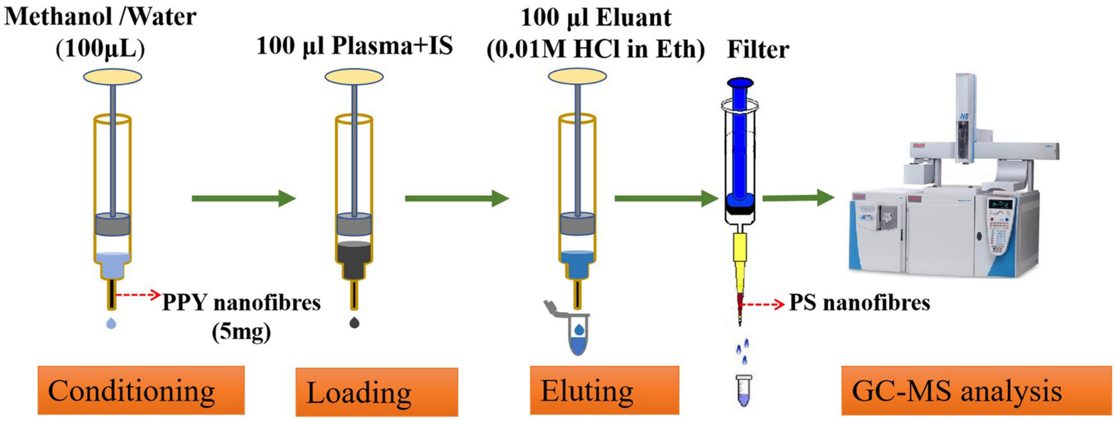

Figure S2: Schematic flow chart of PFSPE procedure for the determination of SCFAs in plasma.

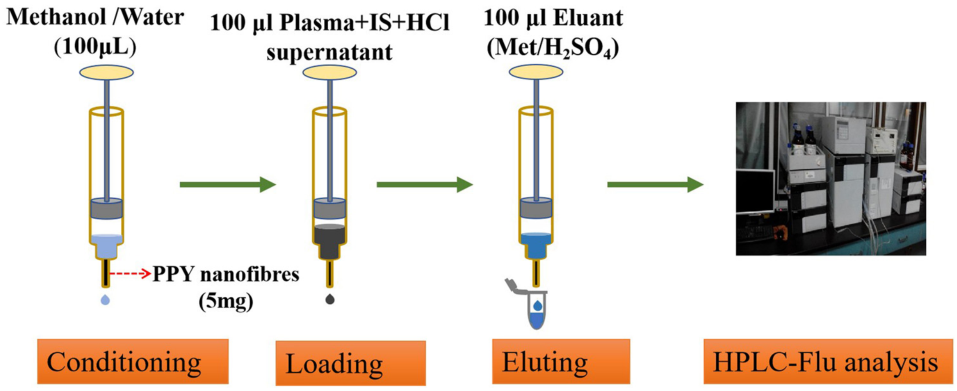

Figure S3: Schematic flow chart of PFSPE procedure for the determination of tryptophan metabolites in plasma.
